# Neural variability, or lack thereof

**DOI:** 10.3389/fncom.2013.00007

**Published:** 2013-02-25

**Authors:** Timothée Masquelier

**Affiliations:** ^1^Unit for Brain and Cognition, Department of Information and Communication Technologies, Universitat Pompeu FabraBarcelona, Spain; ^2^Laboratory of Neurobiology of Adaptive Processes, UMR 7102, CNRS - University Pierre and Marie CurieParis, France

**Keywords:** neural variability, signal-to-noise ratio, reliability, redundancy, neural coding

## Abstract

We do not claim that the brain is completely deterministic, and we agree that noise may be beneficial in some cases. But we suggest that neuronal variability may be often overestimated, due to uncontrolled internal variables, and/or the use of inappropriate reference times. These ideas are not new, but should be re-examined in the light of recent experimental findings: trial-to-trial variability is often correlated across neurons, across trials, greater for higher-order neurons, and reduced by attention, suggesting that “intrinsic” sources of noise can only account for a minimal part of it. While it is obviously difficult to control for all internal variables, the problem of reference time can be largely avoided by recording multiple neurons at the same time, and looking at statistical structures in relative latencies. These relative latencies have another major advantage: they are insensitive to the variability that is shared across neurons, which is often a significant part of the total variability. Thus, we suggest that signal-to-noise ratios in the brain may be much higher than usually thought, leading to reactive systems, economic in terms of number of neurons, and energy efficient.

## Introduction

Randomness is only a measure of our “ignorance of the different causes involved in the production of events” (Laplace, 1825).

High trial-to-trial variability in response to repeated presentation of a same stimulus has been reported in every modality. It is often quantified in terms of reliability and precision (Box 1), and both are usually poor *in vivo* (e.g., Fano factors ~1 and precision ~tens of ms or above). The origin of this variability, and its implication for information processing, has been much debated (Stein et al., 2005; Ermentrout et al., 2008; Faisal et al., 2008; Tiesinga et al., 2008; Rolls and Deco, 2010), yet a consensus has not emerged. Here we argue that most of the observed variability could come from uncontrolled variables, or the use of inappropriate reference times, rather than from intrinsic sources of noise (“intrinsic” meaning that they cannot be eliminated). We focus on sensory systems, where signals are best identified, yet in general not perfectly.

Box 1 **Reliability and precision**

Trial-to-trial variability is often quantified in terms of reliability and precision (Tiesinga et al., 2008). If the same number of spikes is emitted from trial-to-trial, the neuron is said to be reliable. If the timing of such spikes is roughly preserved across trials, the neuron is said to be precise. Reliability is typically estimated using the Fano factor of the spike count on a certain time window, which requires a reference time, that is, its variance divided by its mean. Precision is typically estimated using the spike time dispersion, also called “jitter,” which also requires a reference time. In most cases, the stimulus onset provides this reference time.

## The functional approach: noise, or uncontrolled variables?

Noise is a relative concept. It measures the extent to which a system diverges from its hypothesized, idealized, *function*. For example neurons in early sensory areas are usually hypothesized to encode stimulus features, and only stimulus features. Then trial-to-trial variability in their activity, when controlling for the stimulus, may be called “noise.” If this variability is lower than the variability between different stimuli, then the hypothesis is validated *a posteriori*. Similarly, neurons in primary motor areas are hypothesized to encode motor responses (and only motor responses), and the variability across trials with the same motor response is “noise.”

Unfortunately, neurons' functions are generally unknown, in particular for “higher-order neurons” (that is, farther away from sensory inputs and motor outputs), hence the term “noise” should be used with caution, and the term “unexplained variability” should be preferred. Furthermore, a neuron's function may change over time. For example V1 neurons, when the eyes are closed or in the dark, can be involved in mental imagery (Kosslyn and Thompson, 2003). An experimenter unaware of this will observe a huge unexplained variability in neural activity if he/she fails to control for mental imagery—which is of course difficult and will lead to some variability anyway.

In this paper, we argue that most of the unexplained variability in sensory systems might result from deterministic, but uncontrolled, internal variables mediating attention, degree of arousal, expectations, mental imagery, task-solving strategies, etc. This variability is signal, even though it would look like noise to an experimenter only controlling for the stimulus—all the more so because we know from Shannon's theory of information that when optimal encoding is used to maximize information transmission, neural signals will look random (Faisal et al., 2008). As Barlow wrote about neural responses in 1972, “their apparently erratic behavior was caused by our ignorance, not the neuron's incompetence” (Barlow, 1972).

An extreme case occurs when there are no external variables at all, only (uncontrolled) internal ones, that is when recording spontaneous activity. It should not come as a surprise that trial-to-trial variability is higher in this case than when a same stimulus is repeated, as seen in a number of experiments (Churchland et al., 2010). Indeed, why should activity be the same across trials in which nothing repeats? In some sense, it would be “fairer” to compare spontaneous activity's variability to evoked activity's *without controlling for the stimulus* that is using varied stimuli. Yet of course this approach has flaws too: the results would dependent on how varied the stimuli are.

Since it has a metabolic cost, the spontaneous activity probably has a function. The observed variability might reflect more our inability to grasp it, and to control for appropriate variables, than neurons' unreliability.

## The biological approach: intrinsic and extrinsic sources of variability

Coming back to evoked responses, what mechanisms may cause the commonly observed high trial-to-trial variability? *In vitro*, single neurons stimulated directly by injecting fluctuating currents, in the absence of synaptic input, give highly reliable and (sub)millisecond precise responses (Bryant and Segundo, 1976; Mainen and Sejnowski, 1995; Toups et al., 2012), that deterministic neuronal models can accurately predict (Gerstner and Naud, 2009), despite “channel noise” (Faisal et al., 2008). In a sensory neuronal network, there are two additional intrinsic sources of variability: sensors, which convert physical stimuli into spikes, and synaptic transmission. In many cases, sensors operate close to physical limits that introduce variability (Stein et al., 2005), and therefore should contribute minimally to the variability observed *in vivo*. Synaptic unreliability may have a bigger impact (Movshon, 2000; Faisal et al., 2008). However, high reliability and (sub)millisecond precision is seen in cortex in *some* experiments (see Tiesinga et al., 2008; Haider et al., 2010; Kayser et al., 2010; Panzeri et al., 2010; Herikstad et al., 2011, and references therein), suggesting that it is possible for the brain to overcome this source of variability (Mainen and Sejnowski, 1995), most probably because it is largely independent across synapses, and thus averaged out when a neuron integrates from many of them (we will come back to this point).

So why is trial-to-trial variability so high in other experiments? It could be because: (a) neurons' states when presenting the stimulus differ; (b) neurons receive, in addition to controlled bottom-up sensory signals, uncontrolled top-down extrasensory signals. Both of these variability sources are called “extrinsic,” because in principle they could be eliminated by proper control. We call (c) the intrinsic sources of variability reviewed above (sensors, ion channels, and synapses). In the following sections, we try to rule in or out each possible source of variability in the light of recent experimental findings.

## Inter-neuron correlations

Trial-to-trial variability is typically correlated across neurons (Averbeck et al., 2006), a phenomenon sometimes called “correlated noise.” In other words, a significant part of the total variability is often shared across neurons (Churchland et al., 2010). Variability caused by (c) is expected to be largely independent across neuron. (c) is thus largely ruled out (Table 1, first line).

**Table 1:** **Ruling in and ruling out variability sources (a) different states at stimulus onset, (b) top-down extrasensory signals, (c) intrinsic (sensors, ion channels, and synapses)**.

**Experimental finding**	**Implications for main variability sources**
Inter-neuron correlations	(a) (b) 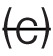
Inter-trial correlations	(a, but only for infraslow oscillations) (b) 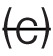
Greater variability for higher-order neurons	(b) ( 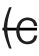 , at least for rapid, feedforward processing)
Attention quenches variability	(a) (b) 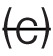

**Figure 1:**
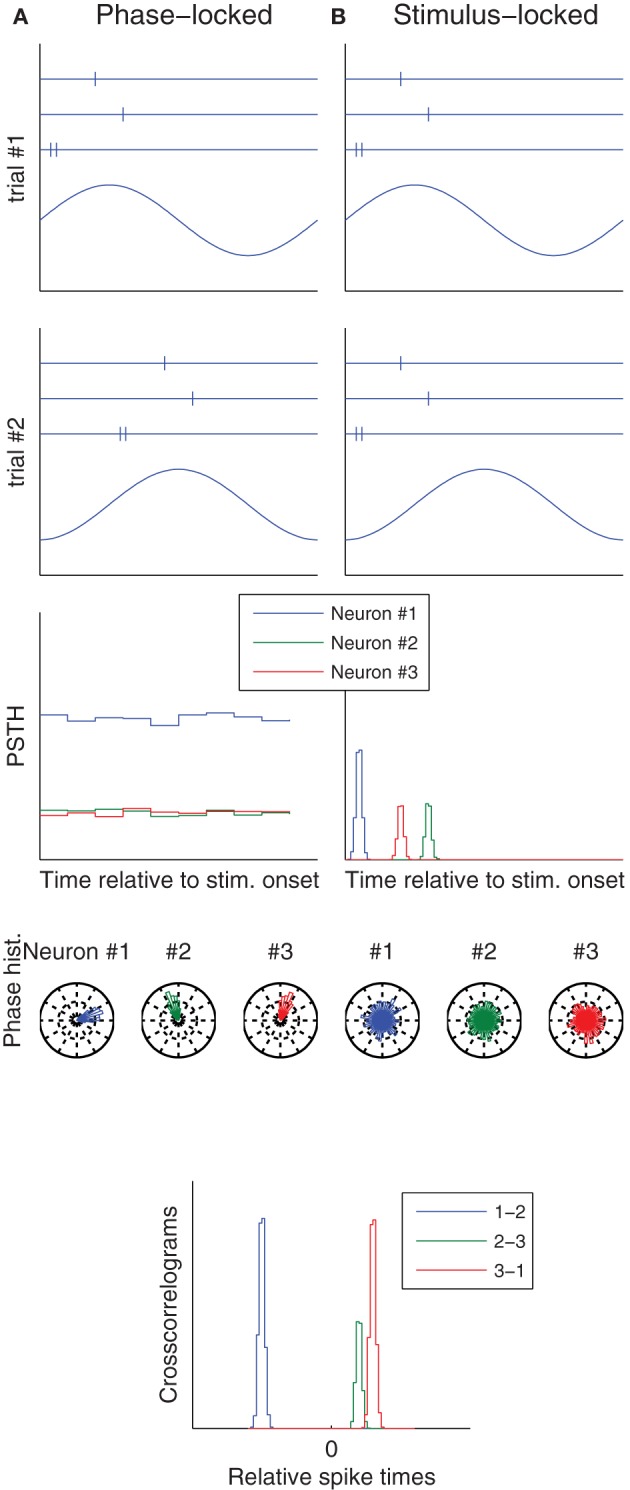
**Phase vs. stimulus locking.** Column **(A)** (resp. **B**) illustrates a situation in which spikes lock to an ongoing oscillation (resp. to stimulus onset). The first two rows correspond to two trials, and show both the raster plots of three neurons (top), and the ongoing oscillation (bottom), whose phase at stimulus onset is different from trial-to-trial. Post-stimulus time histograms (PSTH), which use the stimulus onset as a reference time, only reveal the temporal structure in the stimulus locked-case. Conversely, spike phase histograms, which use the oscillation peak as a reference time, only reveal the temporal structure in the phase locked-case. Spike time cross-correlograms between pairs of neurons reveal the temporal structure in both cases. These are good news, because downstream neurons only care about relative spike times—they ignore both the stimulus onset time and the oscillation phase.

Variability caused by (a) can be correlated across neurons if a common signal determines the neurons' states at stimulus onset. There is much evidence for such signals, which are typically oscillating, and the phase at which a stimulus is presented modulates both evoked neural responses and behavioral performance, in the visual (Vanrullen et al., 2011), auditory (Ng et al., 2012), and somatosensory (Palva et al., 2005) modalities. Therefore (a) is ruled in. Besides, it is worth mentioning that spikes may to lock to these internal oscillations, not to the stimulus onset (Izhikevich, 2006; Tiesinga et al., 2008; Masquelier et al., 2009b; Panzeri et al., 2010). Therefore, using the stimulus onset as a reference time when computing Fano factors or spike time dispersion is inappropriate: it would lead to high values that do not reflect the real reliability and precision (Figure 1).

Variability caused by (b) will be often correlated across neurons as well: top-down signals will typically target multiple neurons and influence similarly their activity. For example in the visual system spatial attention will target all neurons whose receptive fields are in the attended region. Feature-based attention will target all neurons coding for a same feature. Top-down signal are also hypothesized to encore prior expectations, used in a Bayesian inference process (see Chikkerur et al., 2010, and references therein), which will also tend to be similar for neurons coding for similar features. Mental imagery will typically activate similarly all neurons representing the imagined thing. So (b) is ruled in.

Finally, are these inter-neurons correlations beneficial, or detrimental? They are often seen as detrimental, because only independent noise can be efficiently averaged out in a population coding framework, that is when a signal's magnitude is estimated by averaging responses across a pool of neurons with similar tuning properties (Averbeck et al., 2006; Cohen and Kohn, 2011), possibly thanks to stochastic resonance (Stein et al., 2005; Faisal et al., 2008) (Box 2). However, we are notoriously imprecise at “absolute” level estimations if stimuli are presented one at a time (Miller, 1956). We are much better at comparing simultaneously presented stimuli (Stewart et al., 2005). It is likely that we do so by comparing different neurons' activities. In this case, inter-neurons correlations are not detrimental, and even preferable to independent variability: from trial-to-trial, activities will tend to be all shifted in the same direction, preserving the order, thus the relative judgment.

Box 2 **Stochastic resonance**

In neuronal networks, subthreshold signals are not transmitted—only spikes are. This somehow binarizes signals, which can be either subthreshold (no spike), or suprathreshold (spikes). In principle, the magnitude of a suprathreshold signal can be estimated by averaging the firing rate across a long time window. But in practice, it is often not possible because a decision has to be taken rapidly. In any case, it seems that in the brain, neurons' inputs are subthreshold most of the time (Abeles, 1982; König et al., 1996; Brette, 2012). Hence being able to transmit subthreshold signals would greatly enhance the bandwidth. With this goal in mind, it has been suggested that adding noise to a subthreshold signal will cause occasional firings, and more often for near-threshold signals. Therefore, the original subthreshold signal can be estimated by averaging firing rates across time, or across neurons receiving the same subthreshod signal (again, this second option, referred to as “population coding,” is more realistic when reactivity is an issue). Importantly, to be efficiently averaged out, the noise has to be independent across neurons. Besides, there is an optimal level of noise: if too weak, the threshold is not reached often enough; if too strong, the response is dominated by the noise; hence the term “stochastic resonance.”

**Figure 2:**
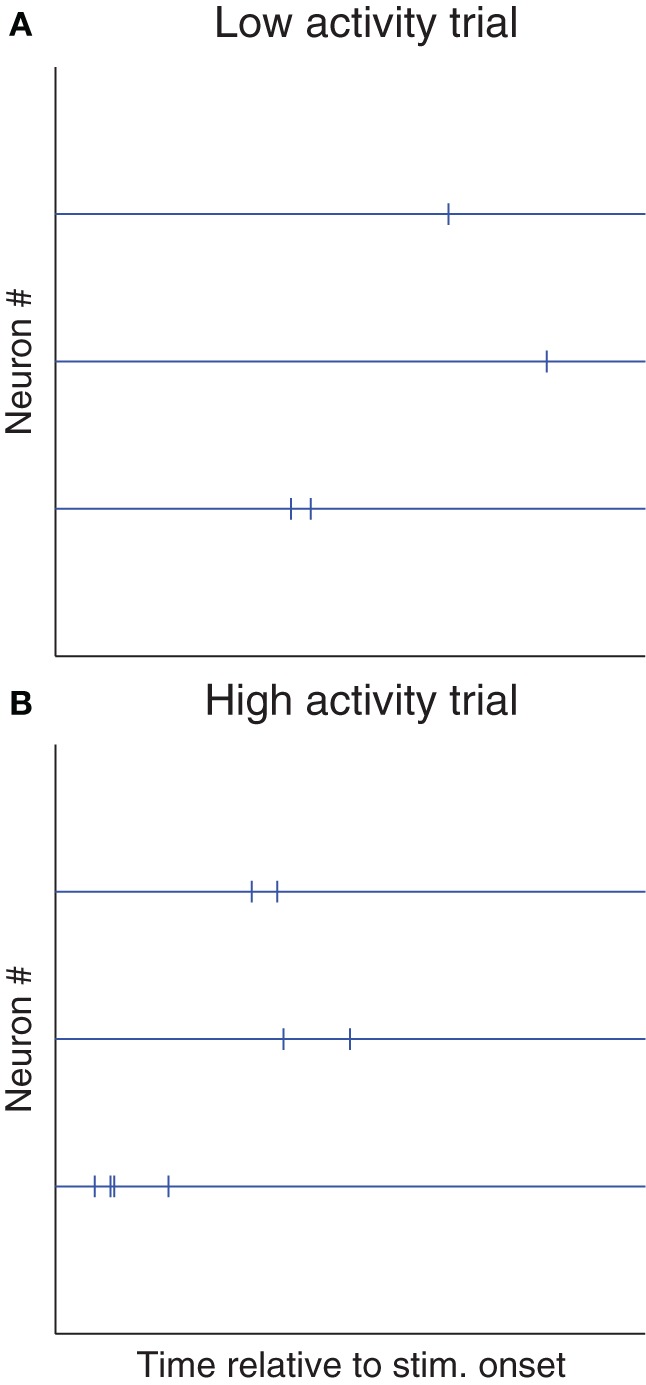
**Shared variability is not detrimental to relative coding schemes.** Here we illustrate a hypothetical situation in which most of the trial-to-trial variability is shared. **(A)** Raster plot of a trial with low spike counts and/or long latencies (for example because the stimulus was presented at a suboptimal phase [source (a)], or because the subject was not attentive [source (b)]. **(B)** Trial with high spike counts and/or short latencies (for the opposite reasons). If these two kinds of trials are observed, Fano factors and spike time dispersion will be high. However, relative spike counts and/or latencies could be more reproducible (because both sources (a) and (b) could affect spike counts and/or latencies similarly across neurons), and could robustly encode the stimulus. Of course, detecting such cases of “relative coding” requires recording multiple neurons at a time, and looking at stimulus-dependent statistical structure in the cross-correlograms. Conversely, neither a PSTH nor a phase histogram (Figure 1) would help.

More specifically, mean spike counts (or latencies), averaged across neurons, could depend on neurons' states at stimulus onset [source (a)], and/or on top-down signals [source (b)], and thus show trial-to-trial variability, while relative spike counts (or latencies) could robustly encode stimulus features (Figure 2). So once again, the observed variability might reflect a wrong assumption of us scientists (stimuli are encoded in absolute spike counts/latencies), more than neurons' unreliability. In line with this proposition, relative latencies have been found to encode stimuli more robustly than absolute ones, in the visual (Desbordes et al., 2008; Gollisch and Meister, 2008; Havenith et al., 2011; Masquelier, 2012; Shriki et al., 2012), somatosensory (Johansson and Flanagan, 2009; Panzeri and Diamond, 2010), auditory (Chase and Young, 2007; Brasselet et al., 2012), and olfactory (Junek et al., 2010; Schaefer and Margrie, 2012) systems.

## Inter-trial correlations

Trial-to-trial variability is often correlated over extended timescales, of tens of seconds or above, which typically involve multiple trials (Monto et al., 2008; Marom, 2010; Marom and Wallach, 2011). What does that tell us about the possible variability sources?

These inter-trial correlations could be caused again by an ongoing oscillation, provided its period is longer than inter-trial intervals (typically a few seconds). There is evidence for such “infraslow” (0.01–0.1 Hz) ongoing EEG oscillations in the somatosensory system, whose phase predicts detection performance (Monto et al., 2008). Therefore (a) is ruled in, but only for infraslow oscillations.

In addition, when an organism is not passively sensing but has to solve a task, these long timescales could be the signature of high-level meta-cognitive processes, in charge of implementing different task-solving strategies, presumably thanks to top-down signals, for example subject-object coupled dynamic exploration (Marom and Wallach, 2011), and changes of these strategies, for example shifting the speed-accuracy or aggressive-conservator tradeoffs, or starting paying more attention to some diagnostic features. Therefore (b) is ruled in.

The mechanisms causing intrinsic variability (sensors, ion channels, and synapses) are commonly thought to have short timescales (<< s). Even though subtle longer term memory effects are sometimes seen (Marom, 2010), they could only account for a very small part of inter-trial correlations. Therefore (c) is largely ruled out.

## Greater variability for higher-order neurons

Variability is typically greater for higher-order neurons. For example in the visual system, both reliability and precision tend to decrease along the ventral pathway (Tiesinga et al., 2008; Herikstad et al., 2011), while top-down effects are greater and greater (Buffalo et al., 2010). Variability is minimal in the retina (Kara et al., 2000; Movshon, 2000), which is out of reach of top-down signals. This is consistent with the proposition that top-down effects are responsible for most of the neural variability. (b) is ruled in. Furthermore, it seems that it is essentially the shared variability, not so much the private one, which increases along the hierarchy: noise correlations are typically low in V1 (Ecker et al., 2010), and higher in extrastriate areas (Faisal et al., 2008). This is again consistent with (b) being a major source of (shared) variability.

To say the same thing in functional terms: higher-order neurons may appear more variable because in general we know less what they are signaling, which may not only be related to the physical stimulus. When by chance we happen to know what a higher-order neuron is signaling, for example a person's identity in case of a so called “grand-mother cell,” or “concept cell,” which selectively responds to photographs of the person as well as his/her written name, then variability is in fact low enough so that this identity can be robustly readout from this sole neuron in a single trial (Quiroga et al., 2005).

How about source (c)? It is estimated that the equivalent of about 50 synchronous excitatory postsynaptic potentials (EPSPs) are required to elicit a postsynaptic spike (Sherwood, 2012). Note that this does not imply redundancy: the 50 presynaptic neurons may be signaling different features, and postsynaptic spikes signal the conjunction of them. But it does imply that independent variability in EPSPs, such as the one caused by (c), will be largely averaged out, and postsynaptic spikes will tend to be more reliable and precise than presynaptic ones. Consequently, spike patterns can be reliably transmitted in feedforward networks, without jitter accumulation (Kumar et al., 2010). This is not consistent with higher-order neurons being more variable, thus (c) is largely ruled out, at least when processing is massively feedforward, that is for rapid sensory processing. When reactivity is less of an issue, the brain can accumulate evidence through recurrent processing (Masquelier et al., 2011). Feedback connectivity makes networks chaotic (Izhikevich and Edelman, 2008; London et al., 2010), that is highly sensitive to small perturbations such as the ones caused by (c), which will end up impacting the whole network. It is thus unclear if higher-order neurons should or should not be more variable in that case, meaning that (c) is neither ruled in, nor ruled out.

It is also unclear if variability caused by different neuronal states at stimulus onset would be greater or lower for higher-order neurons, therefore (a) is neither ruled in, nor ruled out.

## Attention quenches variability

Attention can reduce response (shared) variability (Mitchell et al., 2007, 2009; Cohen and Maunsell, 2009). This rules out a big role for intrinsic sources of noise (c), whereas both sources (a) and (b) are ruled in. The decrease in response variability could be due to some active mechanisms quenching pre-stimulus activity variability, especially when the time-point of the stimulus is predictable (Ledberg et al., 2012), suggesting that (a) might be the main source of variability. Importantly, these drops in variability of both spontaneous and evoked activity lead to improved behavioral performance (Ledberg et al., 2012). Thus, it seems that neural variability is globally detrimental to sensory processing, and that the brain tries to limit it through active mechanisms (we will come back to this point).

## Conclusions

In *some* cases neural responses are both reliable precise, even in cortex (see Tiesinga et al., 2008; Haider et al., 2010; Kayser et al., 2010; Panzeri et al., 2010; Herikstad et al., 2011, and references therein). This suggests that when it is not the case, it might not be because of intrinsic sources of noise, but rather because (1) We did not understand the neuron's function, and thus failed to control for appropriate variables and/or (2) We used an inappropriate reference time, for example the stimulus onset, while spikes locked to an internal ongoing oscillation, or vice-versa (Figure 1). In accordance with these two suggestions, the variability is typically correlated across neurons, across trials, greater for higher-order neurons, and quenched by attention (Table 1).

While it is obviously difficult to control for all extrasensory variables, the problem of reference time can be largely avoided by recording neurons simultaneously, and looking at *relative* spike time statistical structure using cross-correlograms. Relative latencies have another advantage: they are insensitive to the variability shared across neurons (Figure 2), and are thus often less variable than absolute ones (Chase and Young, 2007; Desbordes et al., 2008; Gollisch and Meister, 2008; Johansson and Flanagan, 2009; Junek et al., 2010; Panzeri and Diamond, 2010; Havenith et al., 2011; Brasselet et al., 2012; Masquelier, 2012; Schaefer and Margrie, 2012; Shriki et al., 2012). These are good news, because downstream neurons only care about relative latencies, and the required connectivity to decode them can spontaneously emerge with spike timing-dependent plasticity (Masquelier et al., 2008, 2009a; Gilson et al., 2011; Brette, 2012).

Neurons may thus be more reliable and precise than usually thought, allowing lower redundancy in the brain, that is fewer neurons for a same level of robustness, which is obviously desirable, but also fewer spikes, and thus lower metabolic costs. In line with this proposal, it has been shown that stimulating very few cortical neurons, sometimes only one, and generating only a few extra spikes, can impact behavior (Wolfe et al., 2010). Variability, and thus redundancy, could be particularly low when dealing with suprathreshold stimuli (Gur and Snodderly, 2006)—a more natural situation—and with natural rather than artificial stimuli (Haider et al., 2010; Hasson et al., 2010; Herikstad et al., 2011).

Of course, population coding with redundant noisy neurons would have other theoretical advantages. We have already mentioned stochastic resonance (Box 2). In addition, neuron populations could encode probability distribution over the stimulus, and not only most probable values, and combine them optimally, provided the noise is Poisson-like (Ma et al., 2006). For long processing times (say hundreds of milliseconds or above), noise, or, more accurately, fluctuations, have other benefits: they allow not getting stuck in deadlocks, in a local minima in a minimization problem, or exploring various attractors in multistable perception (Martí et al., 2008; Rolls and Deco, 2010). Importantly, these fluctuations could come from chaotic reverberating activity (Izhikevich and Edelman, 2008; London et al., 2010), exacerbating potentially very weak intrinsic noise. Consistent with this idea, the beginning of responses—which is mostly shaped by feedforward inputs—is typically less variable than the rest of it—shaped by feedback as well (Amarasingham et al., 2006; Churchland et al., 2010).

But despite these potential benefits, we feel that it is too early to conclude that stochasticity is ubiquitous in the brain, and always essential to its function. In a number of cases, it seems to be a nuisance, that can be reduced by attention (Ledberg et al., 2012) and training (Qi and Constantinidis, 2012; Verstynen et al., 2012)—both might in fact shift the neuron's functions towards ones we understand better, leading to the apparent variability reduction.

### Conflict of interest statement

The author declares that the research was conducted in the absence of any commercial or financial relationships that could be construed as a potential conflict of interest.
